# Parental effects driven by resource provisioning in *Alternanthera philoxeroides*—A simulation case study

**DOI:** 10.3389/fpls.2022.872065

**Published:** 2022-09-07

**Authors:** Lan-Hui Wang, Jing Si, Fang-Li Luo, Bi-Cheng Dong, Fei-Hai Yu

**Affiliations:** ^1^School of Economics and Management, Beijing Forestry University, Beijing, China; ^2^Department of Arts and Crafts, Wuhan No. 2 Vocational Education Center School, Wuhan, Hubei, China; ^3^School of Ecology and Nature Conservation, Beijing Forestry University, Beijing, China; ^4^The Key Laboratory of Ecological Protection in the Yellow River Basin of National Forestry and Grassland Administration, Beijing Forestry University, Beijing, China; ^5^Zhejiang Provincial Key Laboratory of Plant Evolutionary Ecology and Conservation, Taizhou University, Taizhou, China

**Keywords:** *Alternanthera philoxeroides*, clonal plant, individual and population scales, mathematical modeling, parental N effects

## Abstract

Parental environmental effects can be a rapid and effective means for clonal plants in response to temporally or spatially varying environments. However, few studies have quantitatively measured the ecological significance of parental effects in aquatic clonal plants. In this study, we developed a two-generation (parent-offspring) growth model to examine the parental effects of nitrogen (N) conditions on summed and mean performance of clonal offspring of one wetland species *Alternanthera philoxeroides*. We also examined the role of survival status and developmental stage of clonal offspring in the consequence of parental effects in aquatic clonal plants. Our results indicated direct evidence that (1) there were significant non-linear correlations between the performance of parental plants and initial status of clonal offspring (i.e., the mass and number of clonal propagules); (2) parental N effects on the summed performance of clonal offspring were content-dependent (i.e., there were significant interactions between parental and offspring N effects), while parental effects on the mean performance of offspring were independent of offspring conditions; (3) parental effects mainly occurred at the early development stage of clonal offspring, and then gradually declined at the late stage; (4) the context-dependent parental effects on the summed performance of clonal offspring gradually strengthened when offspring survival was high. The mathematical models derived from the experimental data may help researchers to not only deeply explore the ecological significance of parental environmental effects in aquatic clonal plants, but also to reveal the importance of potential factors that have been often neglected in empirical studies.

## Introduction

Clonal plants, that spontaneously produce offspring *via* vegetative reproduction, are widespread in nature (de Kroon and van Groenendael, 1997). During the entire life cycle of clonal plants, clonal offspring ramets are repeatedly produced by parental ramets, so the performance of offspring ramets is greatly influenced by the environments that parental ramets have encountered (Latzel and Klimešová, 2010; Douhovnikoff and Dodd, 2015). An increasing body of evidence has documented that parental environments may to some extent regulate the survival, early development, and subsequent growth of clonal offspring across vegetative generations, and also adjust the life-history strategy of clonal offspring to pre-adapt to future environments (González et al., 2016, 2017; Münzbergová and Hadincová, 2017; Dong et al., 2019a,b; DuBois et al., 2020; Huber et al., 2021). Compared to sexually reproducing plants, such parental environmental effects are especially important for clonal plants with a low potential for adaptation through genetically based natural selection.

There are two commonly known types of mechanisms that mediate parental environmental effects in clonal plants, including either the epigenetic-based mechanism such as DNA methylation and histones modification (Latzel and Klimešová, 2010; Douhovnikoff and Dodd, 2015), or provisioning of resources in vegetative propagules such as carbohydrate- and/or nitrogen-based compounds (Dong et al., 2019a; DuBois et al., 2020). For clonal plants, the epigenetic-based mechanism may allow clonal offspring to maintain a long-term and stable phenotype in response to the predictable environments that parental plants have experienced, *via* the accumulation of gene expressions across multiple clonal generations (Douhovnikoff and Dodd, 2015). Alternatively, the changing in provisioning of resources in vegetative propagules caused by parental environments is considered a direct means for clonal offspring in response to the changing environments. Also, the impact of provisioning of resources is expected to become more prominent within several or few clonal generations, because it can directly influence the initial status of vegetative propagules and the sequential growth trajectory of clonal offspring (Dong et al., 2019a; DuBois et al., 2020). From the perspective of mathematical modeling, the parental effects regulated by the provisioning of resources appear to be easily parameterized and accurately estimated in the mathematical models, compared to the parental effects through epigenetic inheritance. However, to our knowledge, studies of quantifying such parental effects have been very scarce.

Several potential factors may influence the magnitude of parental effects in the next clonal generations. The primary factor is the resource level of environments that clonal offspring experienced (Engqvist and Reinhold, 2016; Bonduriansky and Crean, 2018; Yin et al., 2019). One likely scenario is that parental effects will be favorable when the environmental predictability between parent and offspring environments prevails, thereby being often adaptive for clonal offspring (Latzel and Klimešová, 2010; Douhovnikoff and Dodd, 2015). A secondary scenario is that parental effects may interact with offspring environments, but not shift their direction, i.e., the benefits from parental favorable environments may amplify or dwindle with the increased suitability of offspring environments. Such phenomena have been reported especially in the abiotic conditions that offspring experienced, such as drought (González et al., 2016), light (Dong et al., 2019b) and nutrient availability (Dong et al., 2018a). Also, in some cases, parental effects on offspring performance are parallel with the effects caused by offspring environments (Schwaegerle et al., 2000; Dong et al., 2018b).

In addition, the magnitude of parental effects may fluctuate at the different developmental stages of clonal offspring (Schwaegerle et al., 2000; Huber et al., 2021). Provided that parental effects are mainly regulated by the provisioning of resources, such kind of parental effects are predicted to play a key role for clonal offspring at the early developmental stage than at the late stage (Schwaegerle et al., 2000). This speculation may be reasonable that the sufficient supply of resources from vegetative propagules not only guarantees the early normal growth of clonal offspring, but also supports the new development of nutrient absorbing organs such as leaves and roots (Stuefer and Huber, 1999; Dong et al., 2010, 2011; Song et al., 2013). Such early-stage advantage will be gradually weakened at the late developmental stage, especially when the resource supply for plant growth begins to shift from the provisioning of resources from storage organs to the acquisition of the resources from external environments. However, to our knowledge, the changes in the strength of parental effects at different developmental stages have rarely been explored.

Furthermore, the magnitude of parental effects may vary with the study scale of clonal offspring (Dong et al., 2018a). Compared to the performance of individual offspring ramet, the performance of one offspring generation appears to be a complex process, which is closely associated with the survival status, initial-size distribution and number of clonal offspring within one offspring population (Dong et al., 2018a). Given that the performance of the offspring generation is jointly determined by the initial size and number of the surviving offspring, the pattern of parental effects at the offspring-generation level become unpredictable, compared to the performance at the individual level. Previous published studies, indicate that the parental nutrient effect in *Alternanthera philoxeroides* was independent of offspring conditions at the individual level, but also became context-dependent (i.e., the parental nutrient effects interacted with offspring nutrient condition) at the offspring-generation level (Dong et al., 2018a). In an opposite example, because of the trade-off between offspring size and number within one offspring population of the perennial sedge *Scirpus maritimus*, the consequence of parental effects at the individual level was concealed when the overall fitness of clonal offspring are considered (Charpentier et al., 2012). Therefore, it is worth systematically examining the magnitude of parental effects at different study scales.

To provide an explicit test for the parental environmental effects with the provisioning of resources as a potential mechanism, we developed a mathematical model based on the empirical data from two separated greenhouse experiments on the well-studied, amphibious clonal species *A. philoxeroides.* We manipulated N availability as a key external factor that can significantly increase the provisioning of resources in vegetative propagules of clonal plants. Our study especially focused on the following questions: (1) whether parental effects are mediated by the provisioning of resources in clonal propagules, so that they can be predictable *via* modeling? (2) whether parental effects are influenced by the N conditions that clonal offspring experienced? (3) whether parental effects are influenced by the developmental stages of clonal offspring? (4) whether parental effects are influenced by other status of clonal offspring, such as offspring survival?

## Materials and methods

### Plant species

*Alternanthera philoxeroides* (Mart.) Griseb. is a creeping perennial herb of the Amaranthaceae family, native to South America (Holm et al., 1997). The species is considered one of the most noxious invasive weeds in China. This species can rapidly disperse and colonize both aquatic and terrestrial habitats, thereby causing severe ecological and environmental problems (Wu et al., 2016, 2017). In southern China, the invasive populations of *A. philoxeroides* have been reported to belong to the same genotype (Xu et al., 2003; Ye et al., 2003; Wang et al., 2005). *A. philoxeroides* also mainly relies on clonal growth by producing stem and/or root fragments to achieve offspring recruitment (Jia et al., 2009; Dong et al., 2012, 2019a). Each stem node of *A. philoxeroides* can be naturally and/or incidentally fragmented, and become a physiologically independent unit with the potential to develop into ramets (Dong et al., 2012, 2018a).

In this study, plants of *A*. *philoxeroides* were collected from populations in a riparian agricultural area in Taizhou, Zhengjiang, China (28.87^°^N, 121.01^°^E), on 18–19 May 2011. They were vegetatively propagated for more than 3 years in a greenhouse at Beijing Forestry University.

### Experimental design

#### Greenhouse experiment on growth trajectory

To simulate the growth trajectory of plants of *A*. *philoxeroides*, a greenhouse experiment was conducted for 75 days, from 12th June to 24th September 2015. The average air temperature and humidity during the first experiment, measured by HOBO UX100-003 data loggers (Onset Computer Corporation, Bourne, MA, United States), was 25.00 ± 0.31^°^C and 77.80 ± 1.07% (mean ± SE), respectively.

For this experiment, 80 clonal fragments of *A*. *philoxeroides*, each consisting of a single stem of about 10 cm long with two nodes and an apex, were used for the first experiment. Twenty-four additional, similar fragments were selected and dried to estimate the initial dry mass, which was 22.38 ± 0.90 mg (mean ± SE).

Plants were grown in 500 ml plastic plots (9.5 cm in diameter, 13.2 cm deep) fertilized with a modified Hoagland solution containing 10, 20, 40, and 60 mg N L^–1^ supplied as Ca(NO_3_)_2_, to mimic four different nitrogen conditions that cover a range from limiting to non-limiting amounts for plant growth of *A*. *philoxeroides* (Wang et al., 2017). Because we added Ca(NO_3_)_2_ to different Hoagland solutions to vary the concentrations of N, the concertation of Ca^2+^ changed accordingly. To maintain the same Ca^2+^ concentration in different solutions, we need to add CaSO_4_ to compensate for the missing Ca^2+^ in the solutions with high-level N. Finally, only SO_4_^2–^ was always supplied in surplus, since it was expected to impose a negligible impact on plant growth (Wang et al., 2017; Dong et al., 2019c). The gradients of the modified Hoagland solution can be found in Supplementary Table 1. Nutrient solutions were completely refreshed every 4 days to minimize any cumulative buildup or depletion of nutrients. There were four replicates for each N treatment at each harvest.

Plants were separately harvested at 30, 45, 60, and 75 days after the starting of the experiment. At each harvest, plants were washed with distilled water and divided into leaves, stems and roots. Dry masses of each plant part were weighted after oven-drying at 70^°^C for 48 h.

#### Greenhouse experiment on size distribution

To parameterize the size distribution of clonal propagules in *A*. *philoxeroides*, a second experiment was conducted for 45 days, from 22nd July to 4th September 2018. The average air temperature and humidity was 30.43 ± 0.40^°^C and 35.90 ± 0.99% (mean ± SE), respectively.

For this experiment, 28 clonal fragments of *A. philoxeroides* of similar size as in the first experiment, were used. Plants were also grown in 500 ml plastic plots filled with the modified Hoagland solution containing 10, 20, 40, and 60 mg N L^–1^. There were seven replicates of plants for each N treatment. After the 45-day cultivation, stem nodes per plant were counted, and the aboveground part per plant was then subdivided into single-node stem fragments (i.e., clonal offspring) attached with two opposite leaves and half of both the proximal and distal internodes, irrespectively. Each single-node stem fragment (hereafter referred to as clonal propagules) was then stored in individual envelopes. All clonal propagules of each plant and its roots were oven-dried at 70^°^C for 48 h, and weighed.

#### Simulation models—Variable selection

Using the data from the first experiment, we compared the following six classic growth models: linear, exponential, power, monomolecular, three-parameter logistic, and Gompertz models (see Supplementary Appendix Table 1). We then selected the best-fitted model to simulate the growth trajectory of *A. philoxeroides* (Paine et al., 2012). The criterion of model selection was based on the goodness-of-fit (AICs) *via* the “nls” function of the “stats” package. In the growth models, the N levels of external conditions were included as the proxy of the environmental capacity *K*. Also, we examined the correlation between the total mass of each parental plant and the mean mass of the clonal propagules of each parental plant (Eq. 1), and the one between the total mass of each parental plant and the total number of clonal propagules (Eq. 2).


(1) Np⁢r⁢o⁢p⁢a⁢g⁢u⁢l⁢e=α1*TMp⁢a⁢r⁢e⁢n⁢tβ1

(2) M⁢Mp⁢r⁢o⁢p⁢a⁢g⁢u⁢l⁢e=α2*TMp⁢a⁢r⁢e⁢n⁢tβ2

In which *N*_*propagule*_ is the number of clonal propagules produced by one parental plant, *MM*_*propagule*_ is the mean mass of clonal propagules, TM_*parent*_ is the total mass of one parental plant, α_*(1, 2)*_ and β_*(1, 2)*_ are parameters of each correlation equation, respectively.

Using the data from the second experiment, we compared four candidate size distribution models (i.e., the Normal, Log-Normal, Gamma and Weibull distribution) for the size distribution of the clonal propagules produced by one parental plant. The best-fitted model was selected to simulate the size distribution of the clonal propagules of *A. philoxeroides*. The criterion of the best-fitted distribution model was based on the result from the Cramer-von Mises test with the “gofstat” function of the “fitdistrplus” package (Delignette-Muller and Dutang, 2015). Also, we examined the correlations with the mean mass of clonal propagules and each of the parameters from the size distribution model of clonal propagules (Eq. 3).


(3) $$P\,a\,r\,s=\alpha_{\left(3,4\right)}*M\,M_{p\,r\,o\,p\,a\,g\,u\,l\,e}+\beta_{\left(3,4\right)}$$

In which *Pars* are parameters of the size distribution of clonal propagules, *MM*_propagules_ is the mean mass of clonal propagules, α_*(3, 4)*_ and β_*(3, 4)*_ is the parameter of each correlation equation, respectively.

#### Simulation models—Model construction

**FIGURE 1:**
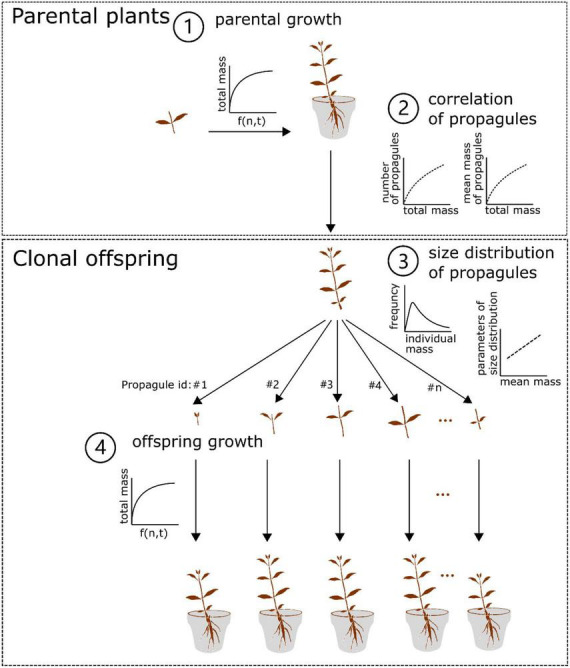
Conceptual diagram of the two-generation growth model of *Alternanthera philoxeroides*. In brief, the parental plant grows following the setup of the three-parameter logistic model with N levels and time included (1), and the final (total) mass of the parental plant is non-linearly correlated with the number and mean mass of its clonal propagules (2). In addition, the size of the surviving clonal propagules produced by the parental plant followed the Weibull distribution, in which the scale and shape values are linearly correlated with the mean mass of the surviving clonal propagules (3). The surviving offspring continue to grow following the setup of the growth model for the parental plant (4). See details in the Material and Method section.

By integrating the growth, size distribution models and the correlations (Eqs. 1–3) as mentioned above, we constructed a two-generation (parent-offspring) growth model (Figure 1). In the model, we assumed that (1) both the parental plants and clonal offspring would follow the growth trajectory as simulated by the same growth model; (2) in the offspring generation, clonal propagules would be totally fragmented and physically independent of one another in homogenous N conditions (i.e., no occurrence of resource sharing and competition between clonal offspring); (3) the size of clonal propagules would be randomly generated by the size distribution model, in which the parameters of the size distribution are regulated by the mean mass of clonal propagules; (4) the offspring derived from clonal propagules with different size would share the same survival rate. In particular, the second assumption seems to be more true for aquatic clonal plants than for terrestrial ones, since aquatic environments are more open and allow for less competition, and resource distribution is also more uniform in aquatic environments. Also, this model derived from the data of stolon fragments may be more valid for the aquatic form of *A. philoxeroides*, which often lacks the formation of taproot system in aquatic environments.

#### Simulation experiment

We used the empirical results from two greenhouse experiments to parameterize the two-generation (parent-offspring) growth model of *A. philoxeroides* (see the conceptual diagram in Table 1). To explore the effect of parental N environments on the performance of clonal offspring at both individual and offspring-generation scales, the parental and offspring N environments were both set along one gradient of N levels (i.e., 10, 20, 30, 40, 50, and 60 mg/L). To explore the parental effects on clonal offspring that experienced different survival status, the survival rates of clonal propagules were separately set as 25, 50, 75, and 100%. One of our interests was to see if the parental effects on the performance of the remaining clonal offspring generation changed, when a number of clonal offspring died due to some other external factors, such as herbivory stress or mechanical removal. It is worth noting that the order of clonal propagules was not considered in the model. To explore the parental effects on clonal offspring at different developmental stages, the growth time of parental plants was constant as 75 days, but the simulated growth time of clonal offspring were separately set as 30, 45, 60, 75, 150, and 300 days. The simulation process was repeated five times. ANOVAs were followed to test the effects of parental N levels, offspring N levels, survival rate, developmental time, and their interactions on the summed and mean performance (i.e., mass) of the surviving clonal offspring produced by one parental plant. All analyses and simulation processes were conducted using R v. 4.0.2 (R Core Team, 2020).

**TABLE 1:** Parameter values used in the simulation experiment.

Parameter/Function	Definition	Values
$\frac{M_{0}\,K}{M_{0}+\left(K-M_{0}\right)\,e^{-r\,t}}$	Three-parameter logistic model (the best-fitted growth model); *M_0* as the initial mass of one plant; *r* as the relative growth rate; *t* as the time of plant growth *K* as the environmental capacity.	*Parental plants*: *M_0* = 22.38 mg; *r* = 0.103; *t* = 75 days. *Clonal offspring*: *r* = 0.103; *t* = 30, 45, 60, 75, 150, and 300 days.
*K* = γ × *N*_*level*_ + δ	*K* in the growth model as a function of N levels.	*Parental plants and clonal offspring*: *N*_*level*_ = 10, 20, 30, 40, 50, and 60 mg/L; γ = 219.642; δ = 1965.396.
Eq. 1	Number of clonal propagules as a function of total mass of one parental plant.	α_1_= 0.253; β_*1*_ = 0.684.
Eq. 2	Mean mass of clonal propagules as a function of total mass of one parental plant.	α_2_= 2.727; β_*2*_ = 0.354.
Weibull distribution	The best-fitted model for the size distribution of clonal propagules produced by one parental plant.	
Eq. 3	Shape value of Weibull distribution as a function of mean mass of clonal propagules.	α_3_= 0.015; β_*3*_ = 0.674.
	Scale value of Weibull distribution as a function of mean mass of clonal propagules.	α_4_ = 1.183; β_*4*_ = −5.083.
Survival rate		25, 50, 75, and 100%.

## Results

### Greenhouse experiments

Within six candidate growth models, the three-parameter logistic model with N levels included was determined as the best-fitted model and used in the following simulation (Supplementary Appendix Table 2 and Supplementary Appendix Figure 1). The corresponding parameter values for the growth model were obtained: *r* = 0.103, γ = 219.642, and δ = 1965.396 (Supplementary Appendix Table 2). In addition, the non-linear regression in Eqs. 1 and 2 fitted the data very well (*R*^2^ = 0.91 and 0.77, respectively; Eqs. 4 and 5 and Supplementary Appendix Figure 2).


(4) Np⁢r⁢o⁢p⁢a⁢g⁢u⁢l⁢e= 0.253*TMp⁢a⁢r⁢e⁢n⁢t0.684

(5) M⁢Mp⁢r⁢o⁢p⁢a⁢g⁢u⁢l⁢e= 2.727*TMp⁢a⁢r⁢e⁢n⁢t0.354

Within four candidate distribution models, the Weibull distribution provided the best-fitting result for the size distribution of clonal propagules in 19 out of the 28 individual parental plants with almost the lowest AICs, thereby being used in the simulation experiment. The scale value of Weibull distribution was well-fitting to the mean mass of clonal propagules by a linear correlation (*R*^2^ = 0.98 and *P* < 0.001; Eq. 6 and Supplementary Appendix Figure 3), and the shape value was marginally significantly linearly related to the mean mass of clonal propagules (*R*^2^ = 0.16 and *P* = 0.088; Eq. 7 and Supplementary Appendix Figure 3).


(6) $$S\,c\,a\,l\,e=1.183*M\,M_{\mathrm{propagule}}-5.083$$

(7) $$S\,h\,a\,p\,e=0.015*M\,M_{\mathrm{propagule}}+0.674$$

### Simulation experiment

#### Number and mean mass of clonal propagules

**FIGURE 2:**
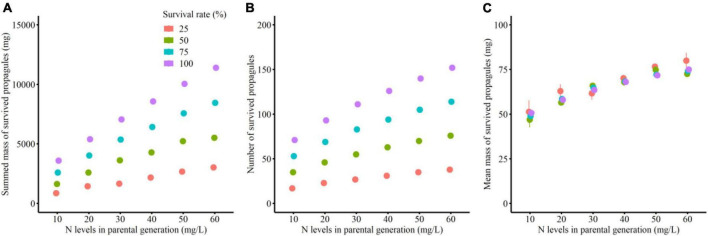
Summed mass **(A)**, mean mass **(B)** and number **(C)** of the surviving clonal propagules produced by each parental plant in the simulation experiment.

Total mass and number of the surviving clonal propagules per parental plant were significantly influenced by parental N levels, survival status of clonal offspring and their interaction (Table 2 and Figures 2A,B). The total mass and number of the surviving clonal propagules were significantly elevated with the increased N levels, and the positive effect of parental N levels was more remarkable when the survival rate of clonal propagules was higher (Table 2 and Figures 2A,B). By contrast, the mean mass of the surviving propagules was independently affected by offspring N levels, rather than by the survival status of clonal propagules and their interaction. The mean mass of the surviving propagules was significantly elevated with the increased N levels, but the positive effect of N levels only tended to be improved when the survival rate of clonal propagules became higher (Table 2 and Figure 2C).

**TABLE 2:** Effects of parental N levels and the survival rate of clonal offspring on the total and mean mass, and the number of the surviving clonal propagules produced by one parental plant.

Variables		Total mass of propagules		Number of propagules		Mean mass of propagules	
	*df*	*F*	*P*	*F*	*P*	*F*	*P*
Parental N (PN)	1, 116	**7,098**	**<0.001**	**8,549**	**<0.001**	**348.0**	**<0.001**
Survival rate (SR)	1, 116	**11,261**	**<0.001**	**30,129**	**<0.001**	3.1	0.079
PN × SR	1, 116	**1,277**	**<0.001**	**1,643**	**<0.001**	1.1	0.293

Degrees of freedom (df), F- and P-values are given. Significance is highlighted in bold.

#### Growth performance of clonal offspring

**FIGURE 3:**
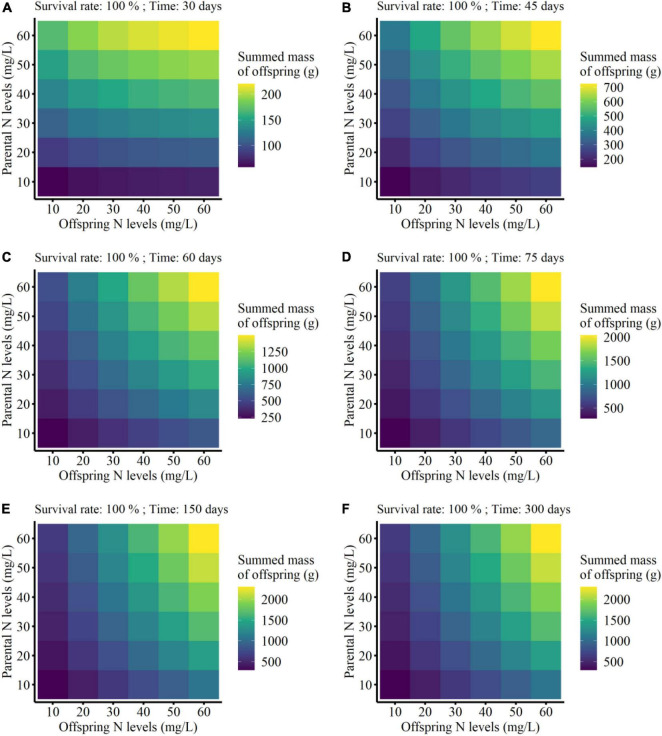
Summed finial mass of the offspring grown from the surviving clonal propagules produced by each parental plant at the different developmental time (from 30 to 300 days; **A–F**) in the simulation experiment. The summed performance of clonal offspring with 100% survival rate were shown here.

The summed mass of clonal offspring was significantly affected by parental N level, offspring N level, survival status, developmental time of clonal offspring, and their interactions (Table 3). Summed performance of clonal offspring significantly improved with the increased N levels in both parental and offspring environments (Figure 3 and Supplementary Figures 1–4). In particular, the benefits from parental high N levels to the performance of clonal offspring became more profound with increased offspring N levels (PN × ON in Table 3 and Supplementary Figures 1–4). The interaction effect between parental and offspring N levels on the summed performance of clonal offspring gradually strengthened with the extended developmental time of plants (PN × ON × T in Table 3 and Supplementary Figures 1–4). In addition, the interaction effect between parental and offspring N levels on the summed performance of clonal offspring gradually strengthened when the survival rate of clonal propagules was maintained at a higher level (PN × ON × SR in Table 3 and Supplementary Figures 1–4).

**TABLE 3:** Effects of parental *N* levels, offspring *N* levels, the survival rate of offspring, and developmental time of offspring on summed and mean performance of clonal offspring produced by one parental plant.

Variables		Summed mass		Mean mass	
	*df*	*F*	*P*	*F*	*P*
Parental N (PN)	1, 4304	**1605.7**	**<0.001**	**17.1**	**<0.001**
Offspring N (ON)	1, 4304	**2754.3**	**<0.001**	**3481.1**	**<0.001**
Survival rate (SR)	1, 4304	**4800.7**	**<0.001**	<0.1	0.753
Time (T)	1, 4304	**2823.3**	**<0.001**	**3670.9**	**<0.001**
PN × ON	1, 4304	**189.8**	**<0.001**	2.5	0.111
PN × SR	1, 4304	**305.1**	**<0.001**	<0.1	0.831
ON × SR	1, 4304	**555.7**	**<0.001**	<0.1	0.904
PN × T	1, 4304	**118.2**	**<0.001**	**6.3**	**0.012**
ON × T	1, 4304	**645.1**	**<0.001**	**834.9**	**<0.001**
SR × T	1, 4304	**577.1**	**<0.001**	<0.1	0.847
PN × ON × SR	1, 4304	**36.0**	**<0.001**	<0.1	0.933
PN × ON × T	1, 4304	**29.6**	**<0.001**	0.8	0.382
PN × SR × T	1, 4304	**23.3**	**<0.001**	<0.1	0.894
ON × SR × T	1, 4304	**131.6**	**<0.001**	<0.1	0.945
PN × ON × SR × T	1, 4304	**5.8**	**0.016**	<0.1	0.962

Degrees of freedom (df), F- and P-values are given. Significance is highlighted in bold.

**FIGURE 4:**
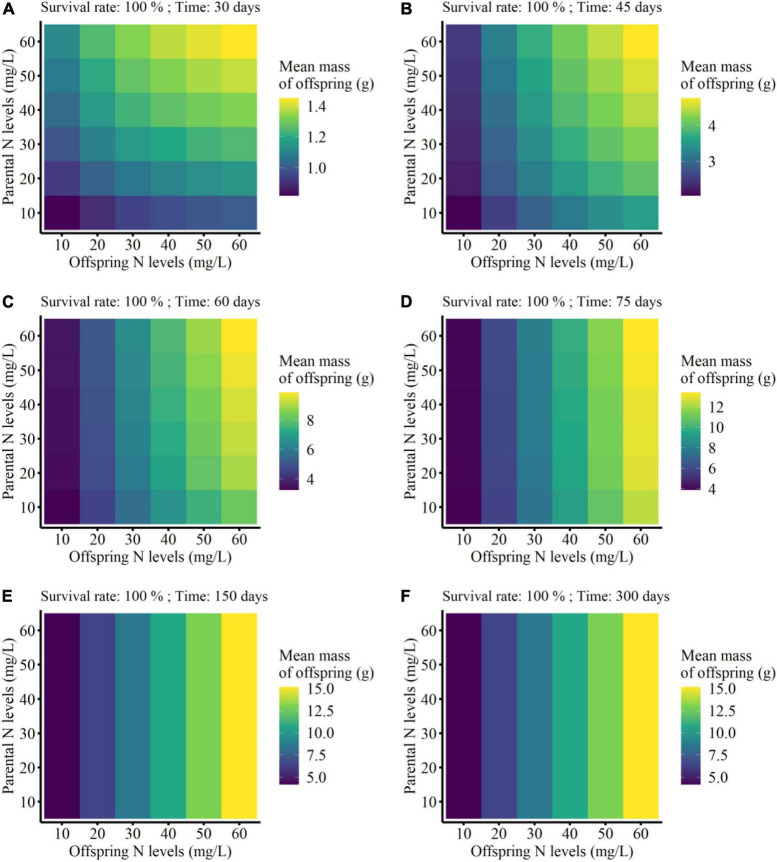
Mean finial mass of the offspring grown from the surviving clonal propagules of each parental plant at the different developmental time (from 30 to 300 days; **A–F**) in the simulation experiment. The mean performance of clonal offspring with 100% survival rate were shown here.

On the other hand, the mean mass of the surviving clonal offspring was only affected by parental N level, offspring N level, and developmental time of clonal offspring, but not affected by the survival rate of clonal offspring (Table 3). Also, there were no interaction effects between survival rate and the other three factors (Table 3). As predicted, the mean performance of clonal offspring improved in the high N levels in both parental and offspring environments. However, the impact of parental N levels mainly occurred at the early stage of plant growth, and then gradually declined and even vanished at the late stage (PN × T in Table 3, Figure 4, and Supplementary Figures 5–8). On the contrary, the impact of offspring N levels persisted at all the stages of plant growth, and then gradually amplified with the time of plant growth increased (ON × T in Table 3, Figure 4, and Supplementary Figures 5–8).

## Discussion

Parental environmental effects are considered a rapid and effective means for clonal offspring to respond to future predictable (stressful and benign) environments that parental plants have often encountered (Latzel and Klimešová, 2010; Douhovnikoff and Dodd, 2015). In this study, we constructed a two-generation growth model to quantify parental N effects mediated by the provisioning of resources. These results from the non-linear correlations between the total mass of parental plants and the mean mass and/or the number of clonal propagules, clearly indicated that there might be a direct relationship between the performance of parental plants and the initial status of clonal offspring (Dong et al., 2019a; DuBois et al., 2020). We speculated that parental effects mediated by the provisioning of resources might be one term of condition-transfer effects, rather than one of anticipating effects (Bonduriansky and Crean, 2018). Such parental effects allowed parental plants to modify the initial status of clonal offspring in response to the ongoing environments, *via* changing the resource investment to vegetative propagule. Such kind of mechanism appears to be very common in the clonal offspring subject to parental abiotic conditions such as temperature, light and nutrient availability (Dong et al., 2018a,2019b; DuBois et al., 2020), and also some biotic conditions such as insect herbivory (Dong et al., 2019a).

Our model has further shown that the outcome of parental effects due to N conditions could interact with the offspring environment. In the present simulation, the positive effect of the parental high-N level was gradually amplified with the increased N levels that clonal offspring experienced. It is also possible for these effects to be some kind of “silver-spoon effect” (Grafen, 1988; Uller et al., 2013; Yin et al., 2019). These parental effects have been previously reported in *A. philoxeroides* (Dong et al., 2018a). The results implied that parental effect could allow clonal offspring to accumulate the size advantage over previous generations in favorable habitats, thereby contributing to the abundance and invasiveness of *A. philoxeroides* in the environments where resource availability is relatively high, e.g., crop fields and irrigation ditches (Pan et al., 2006; Wu et al., 2017).

Besides, the magnitude of parental effects also varied at two study scales (Dong et al., 2018b). The context-dependent parental effect (i.e., the significant interaction effects between parental and offspring N conditions) in *A. philoxeroides* was detected at the offspring-generation scale, but it did not occur in individual performance. These results implied that the context-dependent parental effects might be attributed to some other underlying factors of clonal offspring that was often unrevealed at the individual scale (Dong et al., 2018a). In the simulation experiment, the offspring-size distribution of *A. philoxeroides* followed the rule of the Weibull distribution, in which the shape and scale values of this distribution tended to or strongly correlated with the mean size of clonal propagules. We thus speculated that the parental environments to some degree transformed the size distribution of clonal propagules (e.g., the increased N levels induced clonal offspring to possess a more platykurtic (flat) and symmetrical size distribution with greater mean initial size), so that the majority of clonal offspring within one generation shared the relatively uniform and higher fitness (Dong et al., 2018a). On the one hand, the pattern of skewed offspring-size distribution may strengthen the consequence of parental N effects, and optimize the final performance of the whole offspring-generation especially when the future habitats were further improved (Dong et al., 2018a). On the other hand, the pattern of skewed offspring-size distribution allowed clonal offspring to respond better to temporally or spatially unpredictable environments (Charpentier et al., 2012). In brief, the advantage of skewed offspring-size distribution is not only that more small-sized clonal offspring can grow under favorable conditions, but also that large-sized clonal offspring will keep a strong competitive ability regardless of adverse conditions. In another previous study, the clonal sedge *S*. *maritimus* also employed the variable-size strategy (i.e., the distribution of tuber size followed the log-normal distribution), to adapt to the temporal variation in water levels that characterized its natural Mediterranean environment (Charpentier et al., 2012). Therefore, the selection for the size distribution of clonal propagules may become the often neglected but key factor underlying the consequence of parental effects on the population growth of clonal plants.

The magnitude of the parental N effect varied at different developmental stages of clonal offspring. In the simulation experiment, the early performance of clonal offspring is more susceptible to the parental N conditions, compared to that to the condition that clonal offspring experienced, e.g., the parental N effects were found to be weakened at the late growth period of plants (Schwaegerle et al., 2000). This may be because that the early development of clonal offspring that lacked the mature root system, strongly relied on the provisioning of resources in plant storage organs of parental plants (Dong et al., 2019a; DuBois et al., 2020). When the absorbing organs of clonal offspring (i.e., new leaves and roots) were produced, the clonal offspring may not continue to depend on storage but could attain sufficient resources through assimilation by newly regenerated tissues, so their late development began to be regulated by the ongoing N condition. Therefore, the developmental constraints of clonal plants may play a key role in consequence of parental effects (Yin et al., 2019).

Furthermore, the magnitude of parental N effect at the individual and whole-generation scales was differently influenced by the survival status of clonal propagules. At the offspring-generation scale, the content-dependent parental effects (i.e., parental effects depended on offspring N conditions) interacted with the survival status of clonal propagules. In detail, when the survival rate of clonal propagules within one offspring generation dramatically decreases, the context-dependent parental effects may be to some extent obscured. By contrast, there was no direct association between offspring survival and parental effects at the individual scale. The results further suggested that the reduction in the survival rate of clonal propagules did not only decrease the number of clonal offspring within one population, but also weakened generation expansion of *A. philoxeroides*, especially in resource-rich habitats.

## Conclusion

Our study attempted to quantitatively measure the importance of parental environmental effects in the clonal plants, from the perspectives of individual performance and whole-generation growth. There are several novel findings that have been often neglected in previous studies. First, parental environmental effects could be quantified based on the initial status of clonal offspring (i.e., size of clonal propagules), in the premise that parental effects are mainly regulated by the provisioning of resources, rather than by the genetic and/or epigenetic inheritance. Second, parental environmental effects at the whole-generation scale may be influenced by multiple inherent characteristics of plants (e.g., the survival rate, the number and the size distribution of clonal propagules). Consequently, parental effects differ at the whole-generational level from those at the individual level, as is reflected by the classical paradigm of population ecology. Third, the magnitude of parental effects is to some degree obscured by the developmental constraints of clonal plants. Overall, mathematical models derived from field and/or experimental data may provide some novel perspectives to assist researchers in understanding parental environmental effects in clonal plants.

## Data availability statement

The raw data and main codes required for the analyses are available on GitHub at https://github.com/bichengdong/parental_effect_model.git.

## Author contributions

L-HW and B-CD designed the experiment, did the statistical analysis and the model simulation, and wrote the first draft of the manuscript. JS and B-CD performed the experiment. L-HW, B-CD, F-LL, and F-HY contributed substantially to the revisions. All authors contributed to the article and approved the submitted version.
