# Supplementary material for: Parental effects driven by resource provisioning in Alternanthera philoxeroides—A simulation case study
Source: Front Plant Sci. 2022 Sep 7;13:872065. doi: 10.3389/fpls.2022.872065 (PMC9490186; doi:10.3389/fpls.2022.872065)

Supplementary Materials for

Parental effects driven by resource provisioning in *Alternanthera philoxeroides* - A simulation case study

Lan-Hui Wang, Jing Si, Fang-Li Luo, Bi-Cheng Dong*, Fei-Hai Yu

*Corresponding author. Email: bcdong@bjfu.edu.cn

This PDF file includes:

Supplementary Appendix Tables 1-2
Supplementary Appendix Figures 1-3
Supplementary Table 1
Supplementary Figures 1-8

Appendix Table 1 Forms of six candidate growth models. The model equations are modified from Paine et al. (2012). In the equations, M_0 is the initial mass of the parental plant; *K*: environmental capacity, as a function of N levels and described as $K = \gamma \times N_{level} + \delta$; *r*: the relative growth rate; *t*: the time of plant growth.

Name	Model equation
Linear	$M_0 + rt$
Exponential	$M_0 e^{rt}$
Power law	$M_0^{1-\beta} + rt(1-\beta)^{1/(1-\beta)}$
Monomolecular	$K - e^{-rt}(K - M_0)$
Three-parameter logistic	$\frac{M_0 K}{M_0 + (K - M_0)e^{-rt}}$
Gompertz	$K(\frac{M_0}{K})^{e^{-rt}}$

Appendix Table 2 Fitting results of six candidate growth models, based on the empirical data from the first greenhouse experiment. Power and Monomolecular models failed to converge. Parameters, R^2 and AICs for the fitted growth models are given here. R^2 is R-squared, indicating the goodness of fit for given models; AICs is Akaike information criterion, indicating the relative quality of given models. N/A presents data not available.

Values	Linear	Exponential	Power	Monomolecular	Three-parameter Logistic	Gompertz
r	75.99	0.08	N/A	N/A	0.103	0.02
γ	N/A	N/A	N/A	N/A	219.642	1060.35
δ	N/A	N/A	N/A	N/A	1965.396	8862.27
β	N/A	N/A	N/A	N/A	N/A	N/A
R^2	0.43	0.43	N/A	N/A	0.86	0.86
AICs	1193.43	1181.21	N/A	N/A	1107.22	1109.42

Appendix Figure 1 The three-parameter logistic model with the environmental capacity *K* as a function of N levels, based on the empirical data from the first greenhouse experiment.

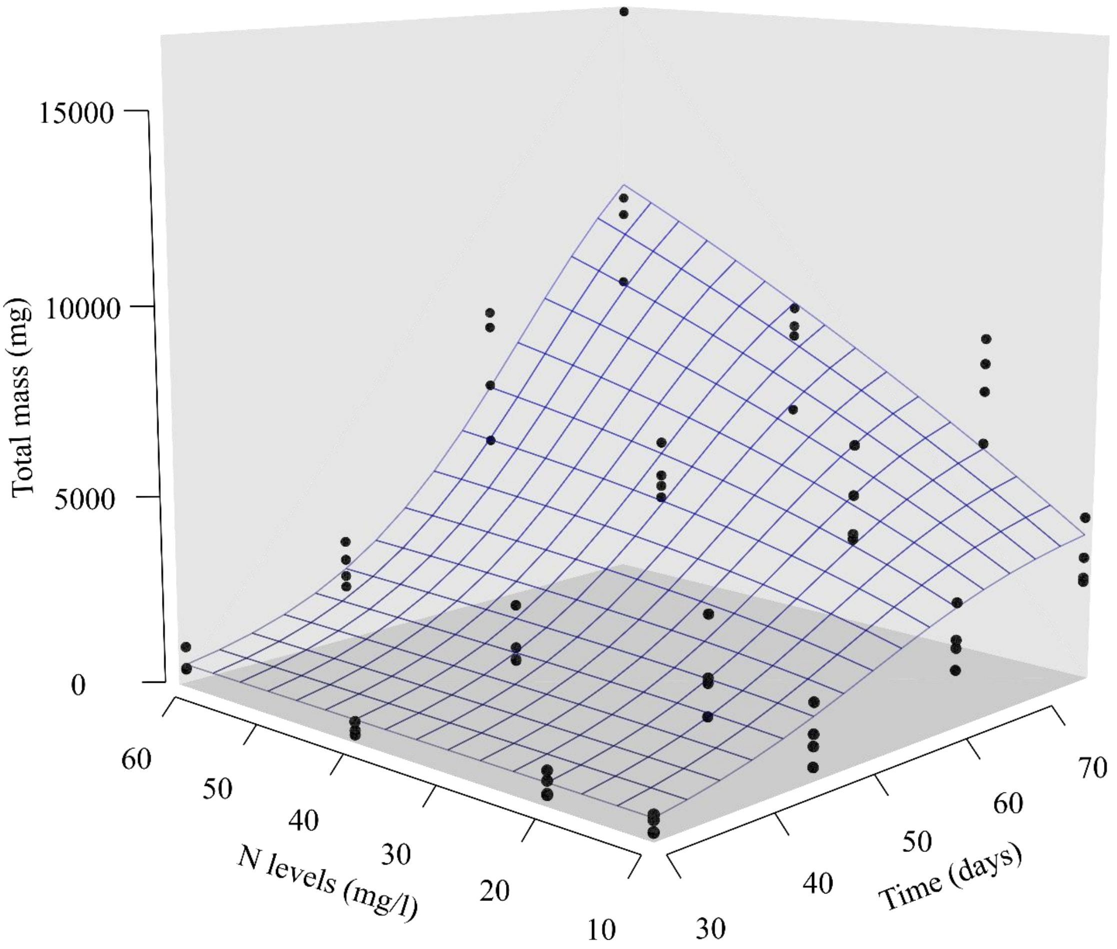

Appendix Figure 2 Non-linear correlations between (A) number of clonal propagules and total mass of each parental plant, and between (B) mean mass of clonal propagules and total mass of each parental plant.

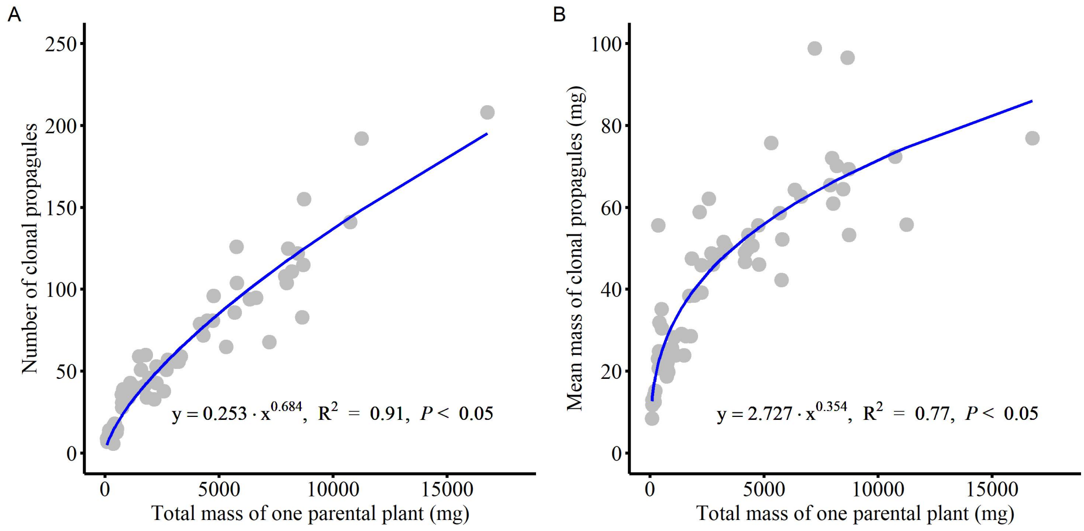

Appendix Figure 3 Linear correlation between (A) mean mass of clonal propagules and shape value of the Weibull distribution that was used in the simulation experiment, and the one between (B) mean mass of clonal propagules and scale value of the Weibull distribution.

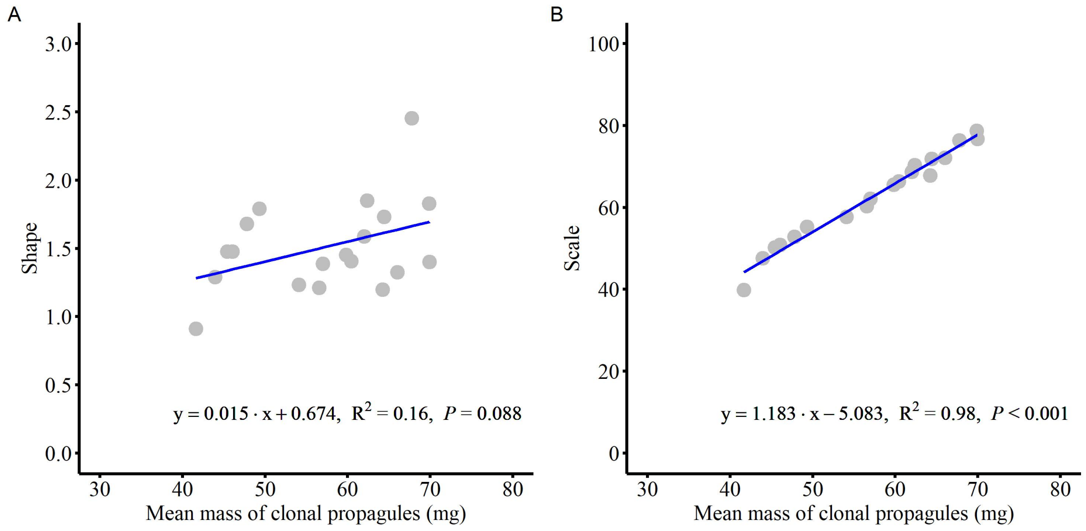

Table 1 Concentration of each composition in the modified Hoagland solution. The concentration of $CaSO_4$ was varied among four N-levels so as to maintain the same total solute concentration except SO_4^{2-}.

Component	Concentration (mg/l)
**Macro-nutrients**	
KCl	111.84
K_2HPO_4	69.67
KH_2PO_4	54.44
$MgSO_4{\cdot}7H_2O$	246.37
EDTA-Fe	34.39
**Micro-nutrients**	
H_3BO_3	2.56
$MnCl_2{\cdot}4H_2O$	1.92
$Na_2MoO_4{\cdot}2H_2O$	0.15
$ZnSO_4{\cdot}7H_2O$	0.44
$CuSO_4$	0.08
$CoCl_2{\cdot}6H_2O$	0.04
**Nitrogen**	
**10 mg/L N**	
$Ca(NO_3)_2{\cdot}4H_2O$	84.23
$CaSO_4$	567.48
**20 mg/L N**	
$Ca(NO_3)_2{\cdot}4H_2O$	168.45
$CaSO_4$	518.92
**40 mg/L N**	
$Ca(NO_3)_2{\cdot}4H_2O$	336.91
$CaSO_4$	421.81
**60 mg/L N**	
$Ca(NO_3)_2{\cdot}4H_2O$	506.04
$CaSO_4$	324.70

Figure 1 Summed finial mass of the offspring grown from the surviving clonal propagules produced by each parental plant at the different developmental time (from 30 to 300 days; A-F) in the simulation experiment. The summed performance of clonal offspring with 25% survival rate were shown here.

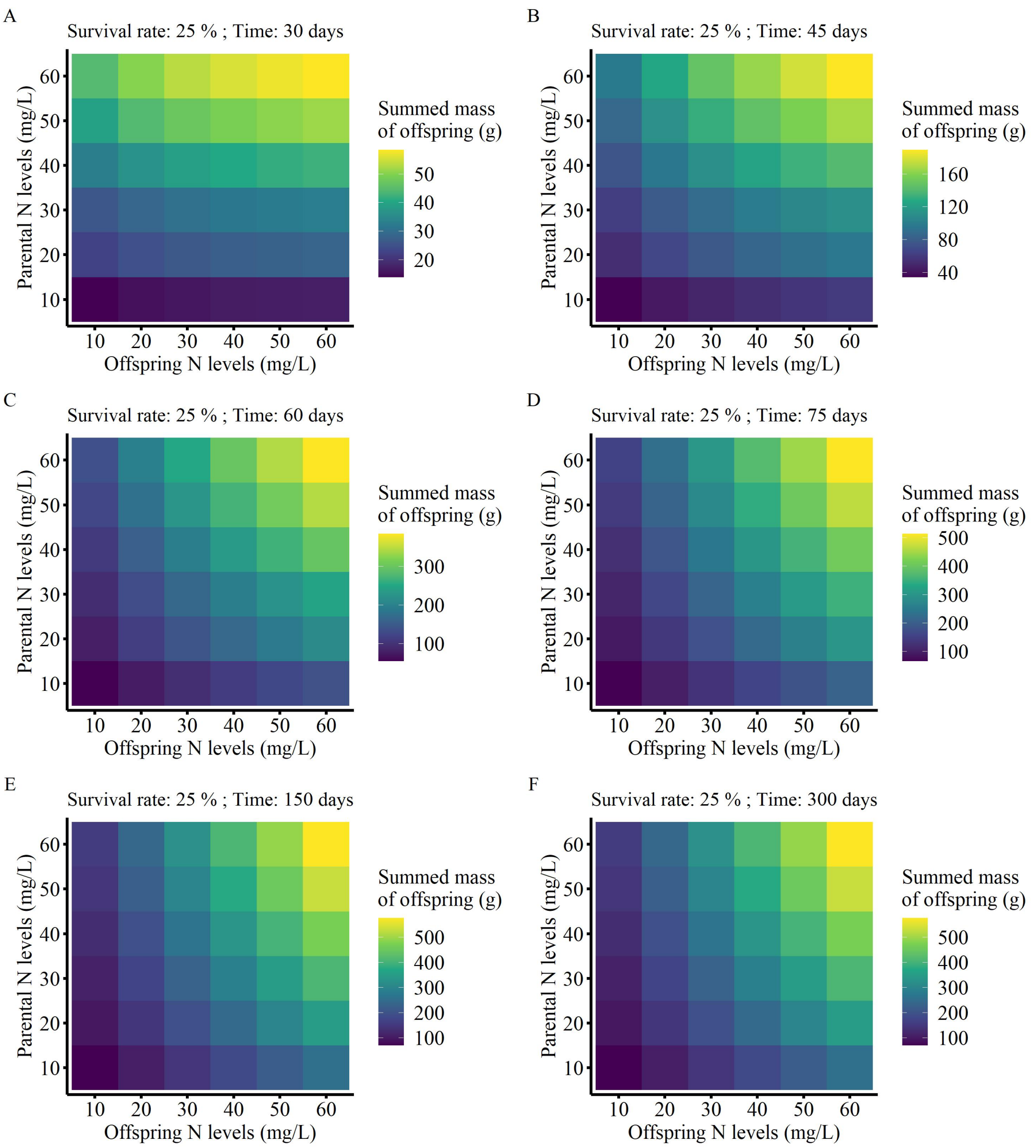

Figure 2 Summed finial mass of the offspring grown from the surviving clonal propagules produced by each parental plant at the different developmental time (from 30 to 300 days; A-F) in the simulation experiment. The summed performance of clonal offspring with 50% survival rate were shown here.

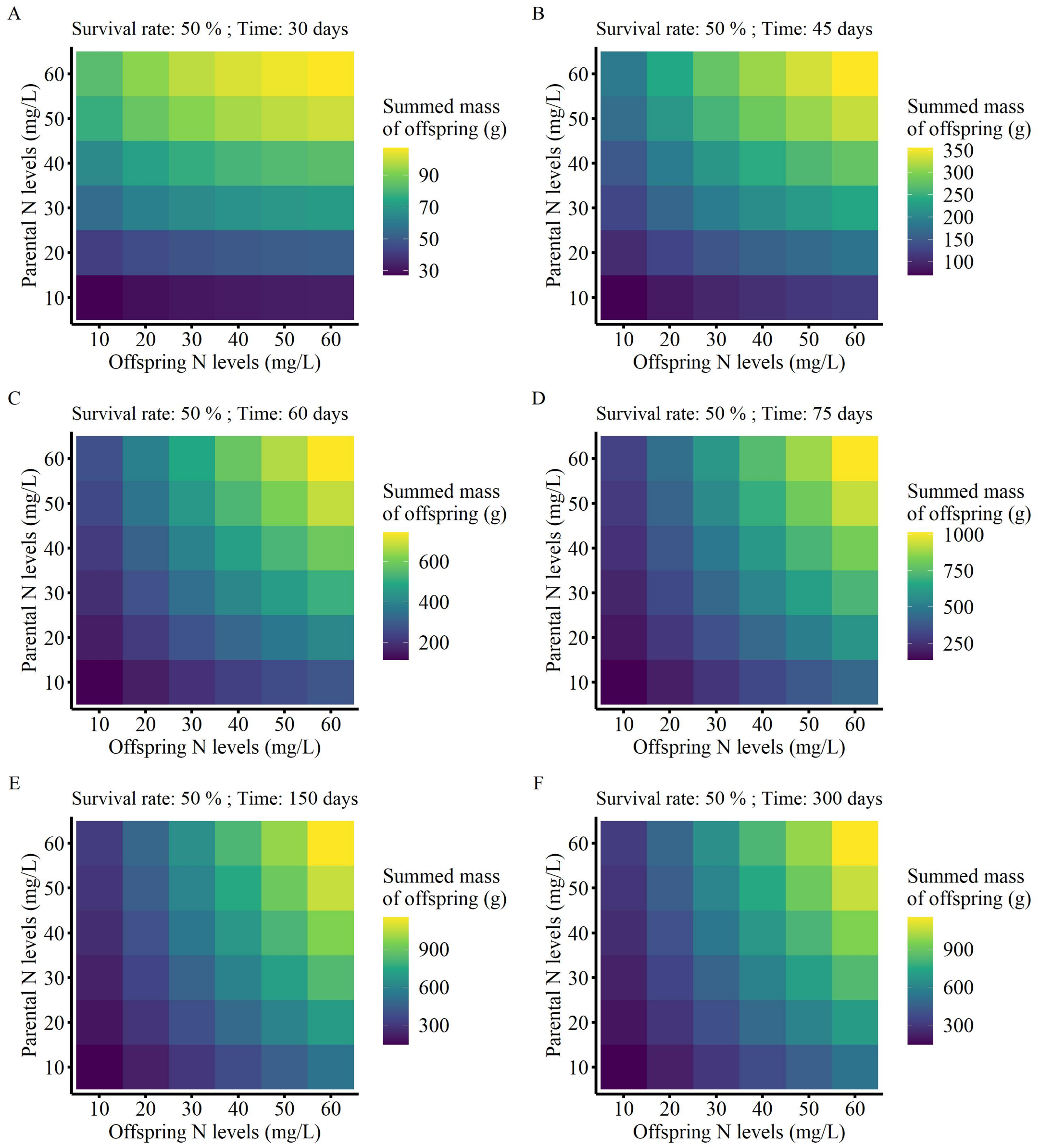

Figure 3 Summed finial mass of the offspring grown from the surviving clonal propagules produced by each parental plant at the different developmental time (from 30 to 300 days; A-F) in the simulation experiment. The summed performance of clonal offspring with 75% survival rate were shown here.

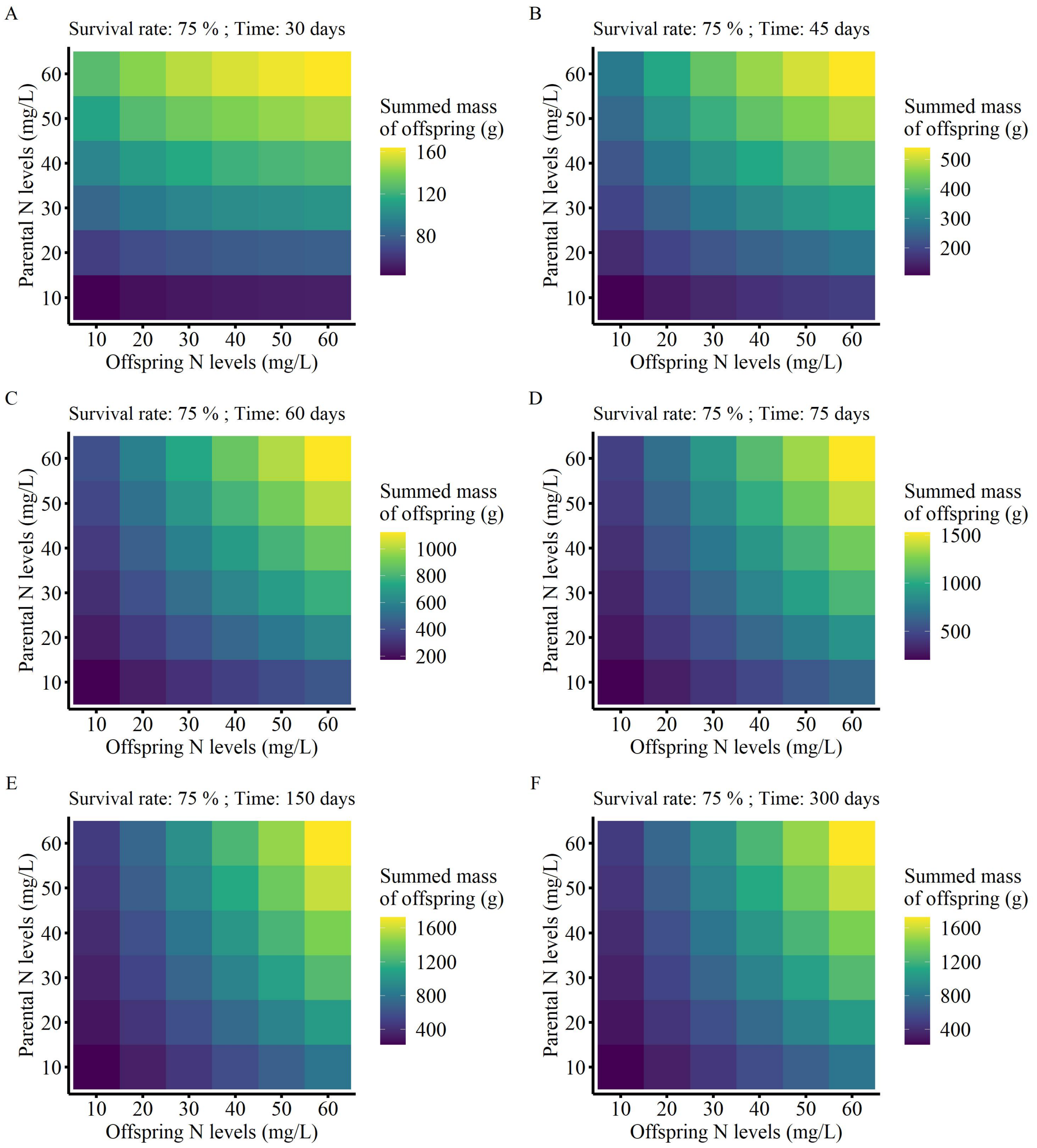

Figure 4 Summed finial mass of the offspring grown from the surviving clonal propagules produced by each parental plant at the different developmental time (from 30 to 300 days; A-F) in the simulation experiment. The summed performance of clonal offspring with 100% survival rate were shown here.

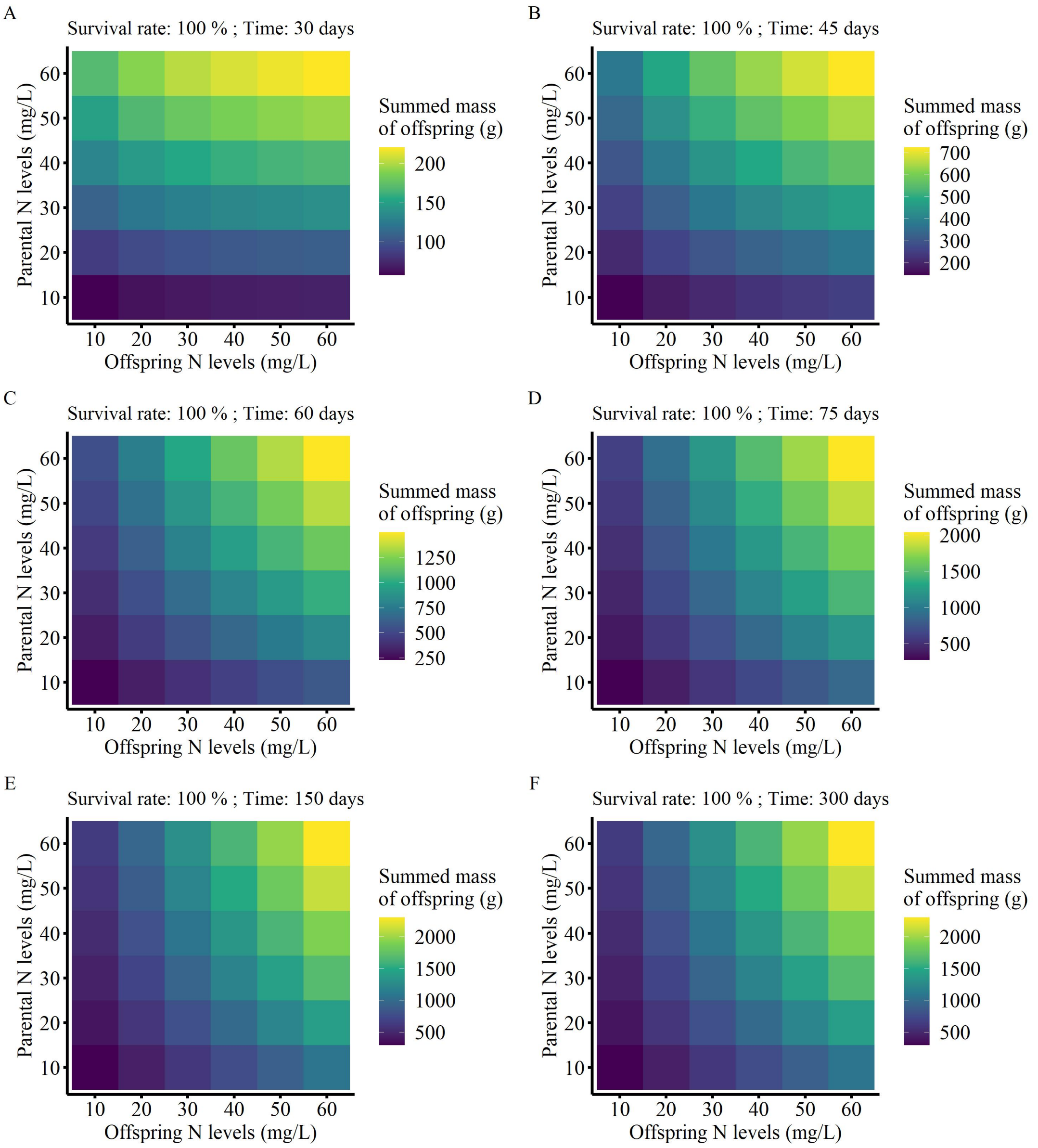

Figure 5 Mean finial mass of the offspring grown from the surviving clonal propagules produced by each parental plant at the different developmental time (from 30 to 300 days; A-F) in the simulation experiment. The mean performance of clonal offspring with 25% survival rate were shown here.

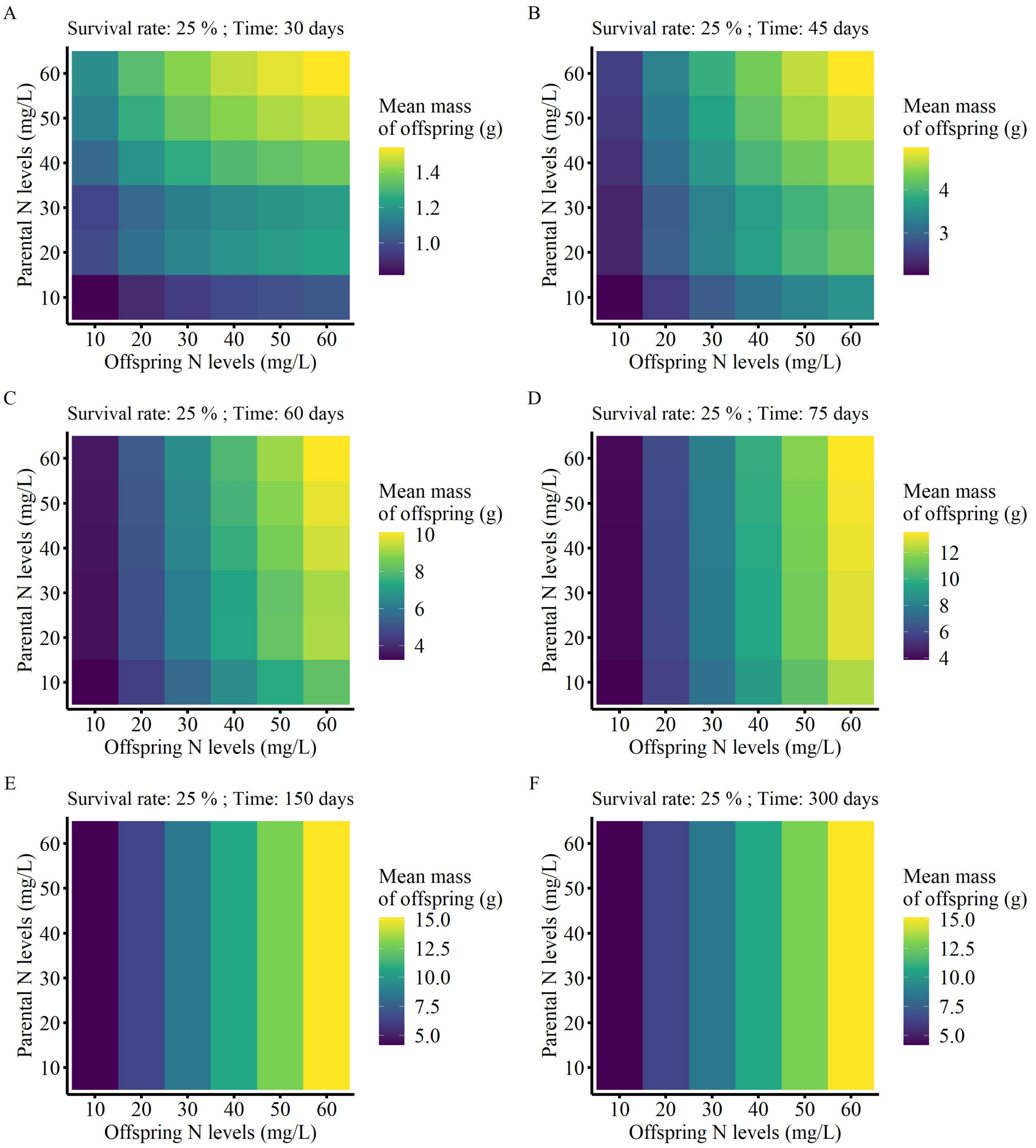

Figure 6 Mean finial mass of the offspring grown from the surviving clonal propagules produced by each parental plant at the different developmental time (from 30 to 300 days; A-F) in the simulation experiment. The mean performance of clonal offspring with 50% survival rate were shown here.

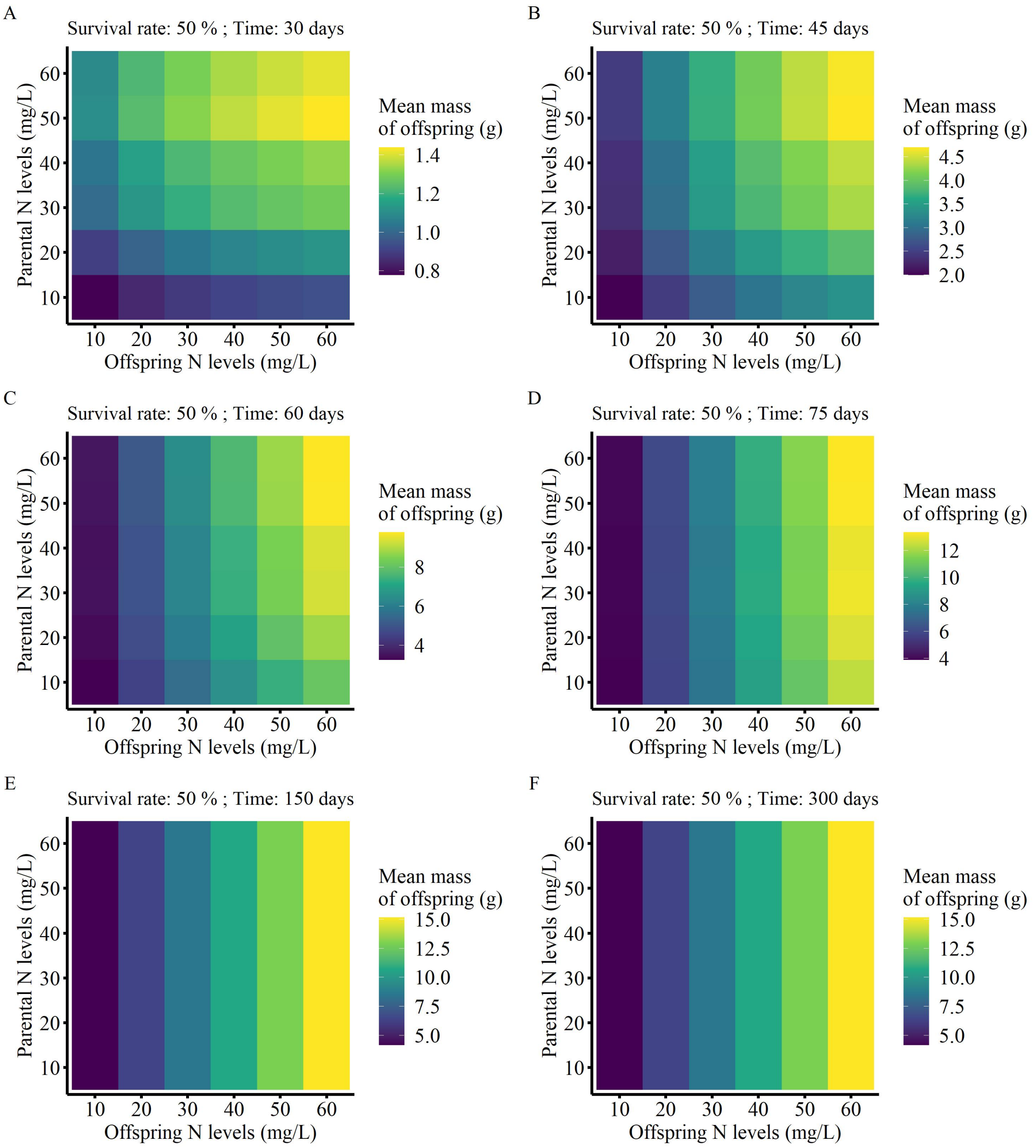

Figure 7 Mean finial mass of the offspring grown from the surviving clonal propagules produced by each parental plant at the different developmental time (from 30 to 300 days; A-F) in the simulation experiment. The mean performance of clonal offspring with 75% survival rate were shown here.

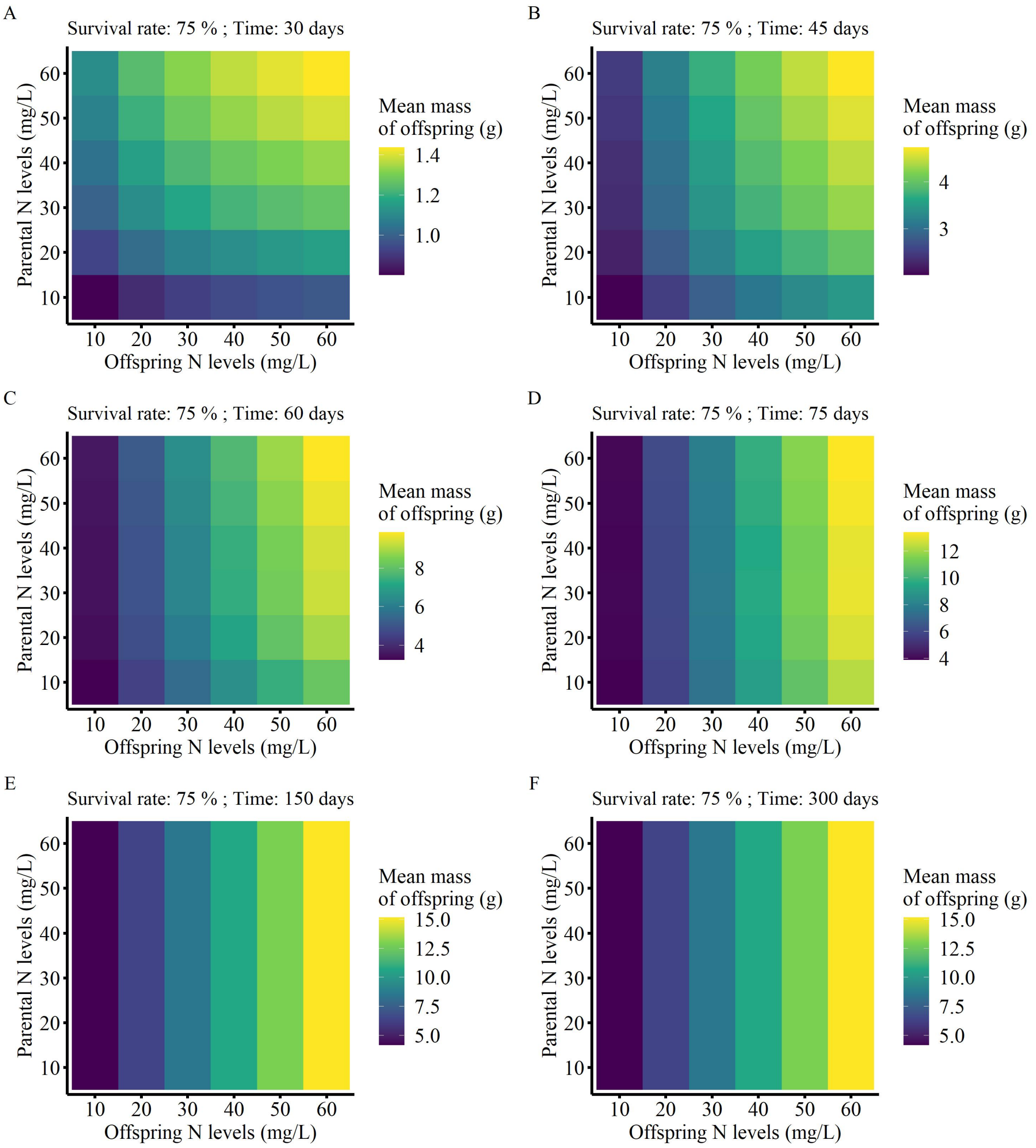

Figure 8 Mean finial mass of the offspring grown from the surviving clonal propagules produced by each parental plant at the different developmental time (from 30 to 300 days; A-F) in the simulation experiment. The mean performance of clonal offspring with 100% survival rate were shown here.

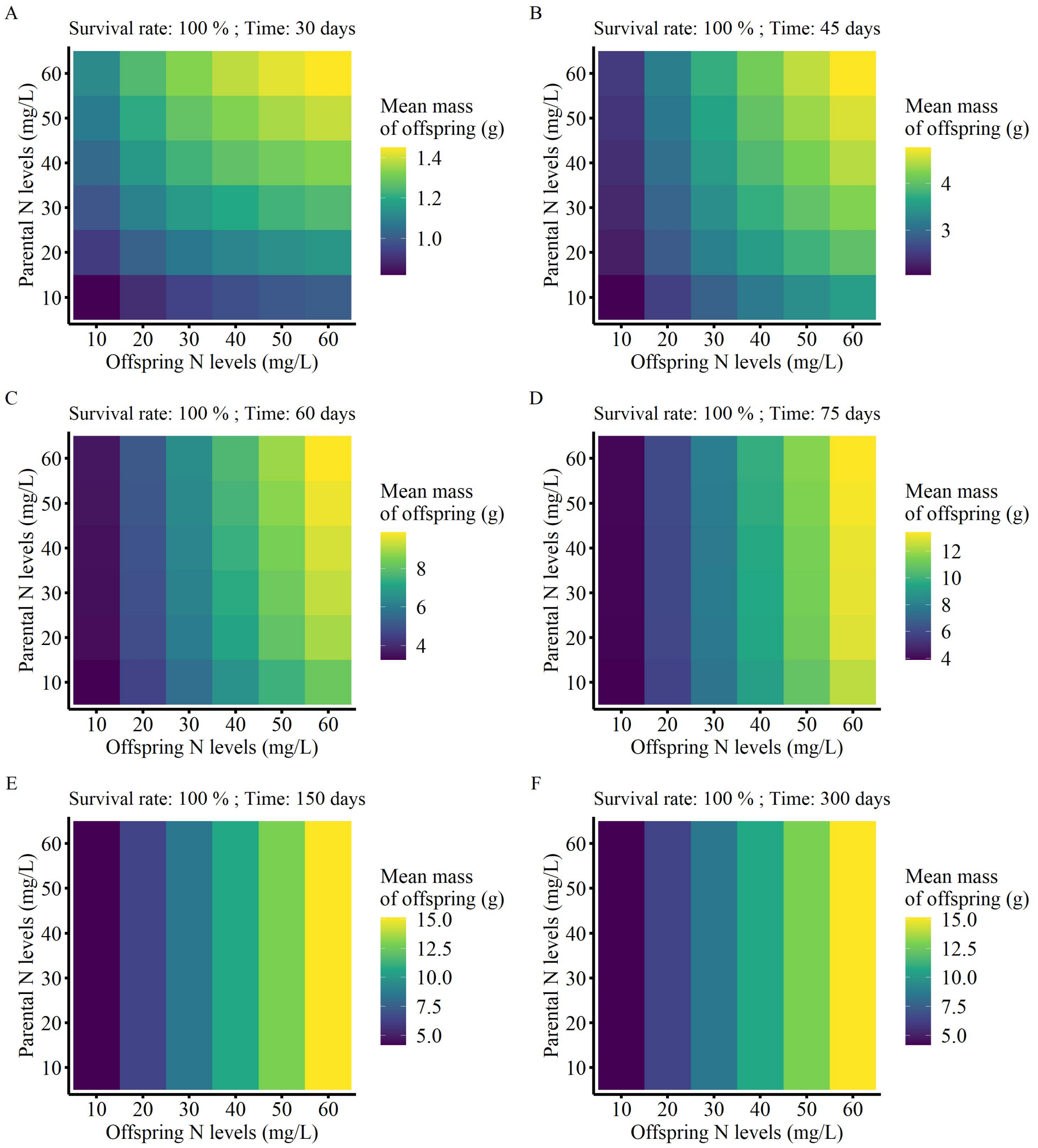

Supplement: Supplementary file 1 [file Data_Sheet_1.pdf]
